# Persistent hypercalcemia with similar familial Hypocalciuric hypercalcemia features: a case report and literature review

**DOI:** 10.1186/s12902-021-00881-9

**Published:** 2021-11-04

**Authors:** Maryam Zahedi, Reyhane Hizomi Arani, Maryam Rafati, Atieh Amouzegar, Farzad Hadaegh

**Affiliations:** 1 Endocrine Research Center, Research Institute for Endocrine Sciences, Shahid Beheshti University of Medical Sciences, Tehran, Iran; 2 Prevention of Metabolic Disorders Research Center, Research Institute for Endocrine Sciences, Shahid Beheshti University of Medical Sciences, P.O. Box: 19395-4763, No. 24, Parvaneh Street, Velenjak, Tehran, Iran; 3 Reproductive Biotechnology Research Center, Avicenna Research Institute, ACECR, Tehran, Iran; 4 Fetal Health Research Center, Hope Generation Foundation, Tehran, Iran

**Keywords:** Familial Hypocalciuric hypercalcemia, Primary hyperparathyroidism, Case report, Endocrinology and metabolism, Persistent hypercalcemia

## Abstract

**Background:**

Primary hyperparathyroidism (PHPT) and familial hypocalciuric hypercalcemia (FHH) are the most important differential diagnosis of parathyroid hormone (PTH)-dependent hypercalcemia. The clinical features of FHH and PHPT can overlap in some cases. Therefore, these two diseases must be differentiated to prevent unnecessary parathyroidectomy. Here, we present a case that was not entirely matched with any of the known differential diagnoses of hypercalcemia.

**Case presentation:**

A 19-year-old girl with no history of any disease presented with persistent hypercalcemia without any specific musculoskeletal complaint. We found persistent hypercalcemia in her routine laboratory data from 3 years ago; while no data was available during the childhood period. Her dietary calcium intake was normal. She did not mention any history of renal stone, bone fracture as well as family history of hypercalcemia. Biochemical features showed normal values of serum creatinine, high normal serum calcium (range, 10.3–11.3 mg/dL; (normal range: 8.8–10.4)), and non-suppressed PTH levels (range, 37.2–58.1 pg/mL; (normal range: 10–65)). Serum 25 OH vitamin D level at the first visit was 16.1 ng/mL that treated by vitamin D supplementation. Since then, all 25 OH vitamin D levels were in the acceptable range. After correction of vitamin D deficiency during the follow-up period the calcium creatinine clearance ratio(s) (CCCR) were calculated in the range of 0.009 to 0.014 (means below 1%). The clinical and laboratory data indicate more FHH rather than PHPT. Genetic studies were negative for the common genes associated with FHH (*CASR, GNA11,* and *AP2S1* genes) and multiple endocrine neoplasia type1 (MEN1). On the other hand, no evidence of autoimmunity was found in her to support an autoimmune FHH-like syndrome. Hence, the case did not match completely to any diagnosis of FHH and PHPT, so we decided to follow her.

**Conclusion:**

We presented a patient with FHH phenotype whose common genetic tests were negative. Further research is needed to ascertain other causes leading to similar manifestations.

## Background

Hypercalcemia has an expanded differential diagnosis of approximately over 25 diseases. Mild-to-moderate hypercalcemia with non-suppressed or high parathyroid hormone (PTH) levels present in patients with primary hyperparathyroidism (PHPT), typically [1]. PHPT is a common endocrine disorder among the general population especially in middle age people and the female gender. Factors favoring PHPT include a history of renal calculi, an asymptomatic patient with a stable prolonged mild hypercalcemia, and rarely the induction of hypercalcemia by thiazides [2]. Familial hypocalciuric hypercalcemia (FHH) and the use of certain medications, such as lithium, could mimic PHPT presentations and should be considered in the differential diagnoses of hypercalcemia with non-suppressed PTH [3].

FHH, also called familial benign hypercalcemia, was initially introduced as a variant of PHPT [4]. It is a rare autosomal-dominant disorder with very low urinary calcium excretion and mild hypercalcemia with high normal or slightly elevated PTH [5]. FHH is a life-long condition that is usually caused by one of many heterozygous inactivating mutations in the calcium-sensing receptor (*CASR*) gene, which could up-regulate the set point of parathyroid cells. When the CASR receptor is inactivated, PTH is not suppressed despite its relatively high calcium, which makes FHH similar to PHPT. In PHPT, although the renal reabsorption of calcium is higher than normal due to the high PTH level, hypercalciuria still occurs [6]. However, in FHH due to CASR inactivation, a general calcium hyposensitivity occurs which results in more tubular calcium reabsorption. So, calcium/creatinine clearance ratio (CCCR = [24 h urine calcium/ total plasma calcium] × [plasma creatinine /24 h urine creatinine]) is lower in FHH compared to PHPT [7].

In addition to familial forms of FHH which existed from the beginning of life, there are other acquired and sporadic forms with unrevealing family screening; including de novo *CASR* gene mutations, and CASR autoantibodies [7]. Patients with CASR autoantibodies mimic the hereditary FHH in biochemical phenotype and could develop FHH-like syndrome. Auto-immune hypocalciuric hypercalcemia is different in pathogenesis from the one caused by inactivating mutations of the CASR [4]. So, a combination of clinical suspicion, biochemical testing, and genetic analysis is needed to distinguish FHH from PHPT. This case is challenging because findings in this case report were not entirely consistent with any of FHH, PHPT, and FHH-like syndrome.

## Case presentation

A 19-year-old girl was referred to our endocrinology and metabolism department with elevated plasma calcium levels found 3 years ago and we had no documentation about it before that. She denied any abnormal complaints including specific musculoskeletal pain, polyuria, polydipsia, fatigue, anxiety, sleep disturbance, loss of concentration and cognitive dysfunction, decreased memory, headache and irritability. Moreover, she had no anorexia, constipation, findings related to pancreatitis, peptic ulcer and abdominal pain. She has had regular menstrual periods since puberty. She did not mention the history of drug use. She had no history of renal stone, bone fracture and cardiovascular disease as well as family history of parathyroidectomy. Her parents had normal calcium levels. Physical examinations were unremarkable. Vital signs and body mass index (BMI: 21 kg/m2) were normal and she never had high blood pressure or orthostatic changes. She had a perfectly normal face. She was quite alert and did not show any signs of depression. No skin lesions were observed. No evidence of muscle weakness, skeletal structural disorders or chondrocalcinosis were observed.

Her serum calcium levels ranged from 10.3 to 11.3 mg/dL (normal, 8.8–10.4, with serum albumin between 4 and 4.2 mg/dL in all assays). Inorganic phosphorus levels ranged from 2.8 to 3.4 mg/dL (normal, 2.5–4.5). Serum magnesium levels ranged from 1.8 to 2.5 mEq/L (normal, 1.7–2.2). Intact PTH levels ranged from 37.2 to 58.1 pg/mL (normal, 10–65) and urine CCCR ranged from 0.009 to 0.014 during these 3 years (Table 1). Serum 25 OH vitamin D level at the first visit was 16.1 ng/mL that treated by vitamin D supplementation. Since then, all 25 OH vitamin D levels were in the acceptable range. Thyroid function tests did not show sub-clinical hyperthyroidism. Other laboratory findings were unremarkable (Table 2).

**Table 1:** Data of Ca, PTH, Vit D and CCCR in the case. (July 2018–January 2021)

	Serum calcium (mg/dL)	Serum phosphorus (mg/dL)	25(OH) Vitamin D (ng/mL)	PTH levels (pg/mL)	CCCR
July 2018	10.8	2.8	–	58.1	–
August 2018	10.8	3	16.1	50.8	0.009
November 2018	10.5	3.3	33.5	40.9	–
December 2018	10.7	3.3	24.7	47.7	0.014
July 2019	10.4	3.2	33.9	–	–
May 2020	10.8	3.4	–	37.2	0.009
June 2020	10.3	3.2	32.5	40.9	–
June 2020	10.6	3.3	–	37.4	0.011
October 2020	11.3	3.3	50.7	–	–
November 2020	11	–	–	–	–
January 2021	–	–	–	45.6	–

*PTH* parathyroid hormone, *CCCR* calcium creatinine clearance ratio.

**Table 2:** Other laboratory findings

Variables	Value	Normal range
White Blood Cells count (WBC) mcL	5600	4.4–11.3 (*1000)
Hemoglobin concentration (Hb)g/dL	14.5	12.3–15.3
Platelet count (Plt) mcL	253,000	336,000 (*1000)
Blood Urea (mg/dL)	25	13–51
Serum Creatinine (mg/dL)	0.64	0.7–1.3
Serum Magnesium (Mg) (mEq/L)	2.1	1.7–2.2
Serum Alkaline Phosphatase (U/L)	50	20–140
Serum thyroid-stimulating hormone (TSH) (mIU/L)	1.16	0.3–4.2
Total T4(μg/dL)	7.0	5.0–12.0
Serum Albumin level (g/dL)	4.1	3.5–5.2
Rheumatoid Factor (IU/mL)	1.4	< 14
Immunoglobulin G4 (mg/dL)	3.65	3–135
Anti-tissue transglutaminase (TTG)-IgA Elisa- Ru/ml	0.1	< 3
Anti-thyroid peroxidase (TPO) IU/mL	0.20	< 35
Immunoglobulin A (mg/dL)	135	70–400
Anti-Nuclear Antibodies-IgG (Dilute)	Negative	

Parathyroid SPECT- CT-MIBI revealed no evidence of parathyroid adenoma. Thyroid, neck, and renal ultrasonography evaluation were normal with no nephrolithiasis or other abnormal findings. On the lateral skull X-ray and both hands AP X-ray, there were no abnormal findings associated with chronic inappropriate high PTH level; including subperiosteal bone resorption, brown tumors, bone cysts, or sclerosis.

Genomic DNA was extracted from the submitted specimen and the Lon AmpliSeq^114^.Exome RDY Library Preparation Kit was used to amplify some targeted regions of gene panel. Among 19,073 known genes sequenced, variant analysis firstly focused on three genes associated with FHH, including *CASR, GNA11*, and *AP2S1*. The further variant analysis was carried out on *MEN1, RET, CDKN1B, GCM2, CDC73*, and other genes associated with PHPT induced hypercalcemia. None of the genetic studies in our case were informative.

Although bone mineral density (BMD) measurement is not appropriate at a young age, it was eventually done, because it may help to differentiate FHH from PHPT [8, 9]. BMD of the femoral neck, lumbar spines, and wrist showed normal values (Table 3).

**Table 3:** Dual-energy x-ray Absorptiometry (DEXA)

Region	Area (cm^**2**^)	BMC(g)	BMD (g/cm ^**2**^)	T-score	Z-score
**Total Lumbar spine**	67.56	70.08	1.037	−0.1	0.2
**Total Hip**	31.73	30.06	0.948	0.0	0.0
**Ultra-distal Radius**	15.30	8.02	0.524	−1.0	−1.0
**10-Year Fracture Risk**					
Major Osteoporotic Fracture	3.0%				
Hip Fracture	0.1%				

## Discussion and conclusion

We have described a young lady with persistent mild hypercalcemia, high normal PTH, CCCR ≤0.01, without any clinical findings of PHPT, and any related family history of hypercalcemia and nephrolithiasis. Genetic studies were negative for known FHH mutations and the *MEN 1* gene. No evidence of the presence of autoimmune FHH like syndrome was detected.

FHH and PHPT are the two most important differential diagnoses in this patient. A two-step diagnostic procedure was used for distinguishing between FHH and PHPT. The first step was the CCCR from a 24-h urine sample. A ratio of less than 0.01 suggests FHH, and a ratio of 0.02 or higher could indicate PHPT. For the second step, all patients with a CCCR of 0.020 or less are tested for mutations in the *CASR* gene. The diagnostic sensitivity of these two steps is 98% [7]. The majority (80%) of patients with FHH have a CCCR of < 0.01; nevertheless, almost 20% of patients with PHPT have a CCCR between 0.01 and 0.02 which can overlap with FHH individuals. A low CCCR may be seen in PHPT with renal insufficiency or in those with severe vitamin D deficiency; the patients have a slightly elevated or high normal serum calcium and a low urinary excretion of calcium [2], while our patient had low CCCR without renal failure and vitamin D deficiency. On the other hand, a large retrospective cohort study was done on 1000 patients with definitive diagnosis of PHPT based on surgery, which showed CCCR is valuable only in 37% patients to correctly differentiate FHH from mild PHPT. In 63% patients with surgically verified PHPT, CCCR was below 2% where both FHH and primary hyperparathyroidism are overlapping [10].

Genetic testing of the *CASR* gene is helpful in situations in which there is an overlap in the phenotype of PHPT and FHH [11, 12]. On the other hand, PHPT is more prevalent among individuals over 50 years, so young patients with PHPT may be suspected of multiple endocrine neoplasia (MEN) [13], which is why we tested the MEN1 gene mutation for our patient.

FHH is not always a familial disease, despite its name; hence, the lack of evidence in family screening does not rule out FHH. It’s important to note that approximately one-third of individuals with FHH have no mutations within the *CASR* gene [8]. It may be due to mutations located outside the coding exons 2–7 of the CASR or mutations which were not detected by PCR and sequencing [7].

Kifor et al. reported several patients with PTH-dependent hypercalcemia with presentations that seemed to be caused by autoimmunity (e.g., non-tropical sprue or Hashimoto’s thyroiditis) [4]. Another acquired hypocalciuric hypercalcemia has been reported in a woman with a severe autoimmune disorder because of IgG4 autoantibodies [14]. Although the presence of CASR antibodies is needed for confirmation of the diagnosis of FHH-like syndrome, in most reported patients with FHH-like syndrome, other obvious autoimmune diseases were also found [4]. In this case, it was not available to check CASR antibodies. However, we can claim that FHH-like syndrome was very unlikely, due to a lack of clinical and laboratory evidence of other associated autoimmune diseases, including non-tropical sprue, Hashimoto’s thyroiditis, Ig G4 related disease, systematic lupus erythematosus, and rheumatoid arthritis.

In our case, the first rank diagnosis was FHH because of young age at the time of hypercalcemia detection, lack of history of fractures, nephrocalcinosis, nephrolithiasis, radiologic suspicion for bone resorption, and a normal BMD as well as CCCR≤0.01 [15]; although these presentations may be present in nearly 20% of patients with PHPT [16]. Also, the absence of autoantibody markers and lack of other autoimmune diseases could rule out FHH-like syndrome. On the other hand, FHH rarity, absence of a family history of FHH, and negative results of relevant genetic tests could support PHPT as the second rank in diagnosis.

Finally, we decided to follow the case rather than referring her to parathyroidectomy, focusing on changes in serum calcium, PTH, and CCCR levels. Also, we plan to repeat BMD at an older age because bone complications could occur in PHPT patients over time, unlike most patients with FHH [17, 18].

Our study has some limitations, including the fact that the CASR autoantibody was not checked in the patient. Also, we did not have any related tests before this period. Finally, although we checked her parent’s laboratory data, we could not access to the exact status of the calcium levels in her brother. Of course, the patient^’^s family mentioned that their son was healthy.

In conclusion, we have described a young lady with FHH phenotype (e.g., persistent mild hypercalcemia, high normal PTH, CCCR ≤0.01), with negative genetic tests. The findings in this case report were not entirely consistent with any of FHH, PHPT, and FHH-like syndrome. Further researches are needed to ascertain other causes leading to similar manifestations.

## Data Availability

All data used during the current study are available from the corresponding author on reasonable request.

## Abbreviations

FHH: Familial Hypocalciuric Hypercalcemia
PHPT: Primary Hyperparathyroidism
CCCR: Calcium creatinine Clearance Ratio
CaSR: Calcium Sensing Receptor
